# Exon duplications in the *ATP7A *gene: Frequency and Transcriptional Behaviour

**DOI:** 10.1186/1750-1172-6-73

**Published:** 2011-11-10

**Authors:** Mie Mogensen, Tina Skjørringe, Hiroko Kodama, Kenneth Silver, Nina Horn, Lisbeth B Møller

**Affiliations:** 1 Center for Applied Human Molecular Genetics, Kennedy Center, Gl. Landevej 7, 2600 Glostrup Denmark; 2 Department of Health and Dietetics, Faculty of Health and Medical Sciences, Teikyo Heise University, 2-51-4 Higashiikebukuro, Toshima-ku, Tokyo 170-8445, Japan; 3 Departments of Pediatrics and Neurology, Comer Childrens Hospital, University of Chicago5841 South Maryland Ave. Chicago, Illinois, USA

## Abstract

**Background:**

Menkes disease (MD) is an X-linked, fatal neurodegenerative disorder of copper metabolism, caused by mutations in the *ATP7A *gene. Thirty-three Menkes patients in whom no mutation had been detected with standard diagnostic tools were screened for exon duplications in the *ATP7A *gene.

**Methods:**

The *ATP7A *gene was screened for exon duplications using multiplex ligation-dependent probe amplification (MLPA). The expression level of *ATP7A *was investigated by real-time PCR and detailed analysis of the *ATP7A *mRNA was performed by RT-PCR followed by sequencing. In order to investigate whether the identified duplicated fragments originated from a single or from two different X-chromosomes, polymorphic markers located in the duplicated fragments were analyzed.

**Results:**

Partial *ATP7A *gene duplication was identified in 20 unrelated patients including one patient with Occipital Horn Syndrome (OHS). Duplications in the *ATP7A *gene are estimated from our material to be the disease causing mutation in 4% of the Menkes disease patients. The duplicated regions consist of between 2 and 15 exons. In at least one of the cases, the duplication was due to an intra-chromosomal event. Characterization of the *ATP7A *mRNA transcripts in 11 patients revealed that the duplications were organized in tandem, in a head to tail direction. The reading frame was disrupted in all 11 cases. Small amounts of wild-type transcript were found in all patients as a result of exon-skipping events occurring in the duplicated regions. In the OHS patient with a duplication of exon 3 and 4, the duplicated out-of-frame transcript coexists with an almost equally represented wild-type transcript, presumably leading to the milder phenotype.

**Conclusions:**

In general, patients with duplication of only 2 exons exhibit a milder phenotype as compared to patients with duplication of more than 2 exons. This study provides insight into exon duplications in the *ATP7A *gene.

## Background

Menkes disease (MD; MIM# 309400) is a multisystemic lethal disorder of impaired copper metabolism due to mutations in the X-linked *ATP7A *gene [1,2]. The disorder is transmitted in an X-linked recessive pattern. The ATP7A protein is a member of the P-type ATPase family that ensures the ATP-driven translocation of metal cations across cellular membranes. The protein plays a dual role: it is responsible for the copper-loading of several copper-requiring enzymes, as well as for the ATP-driven efflux of copper from the cell [3-5]. At normal physiological copper concentrations, ATP7A is localized to the trans-Golgi network (TGN) [3] where copper-loading of enzymes in the secretory pathway takes place. In response to the increase in copper concentration, the protein is translocated to the plasma membrane [3] where it is involved in pumping excess copper out of the cell. In the human body, copper is taken up in the gastrointestinal tract. However, patients with MD are unable to transport copper further from the intestinal cells, and less copper is therefore delivered to the blood. These patients have severe developmental and neurological impairments due to a sub-normal amount of copper in the brain. In addition, a reduced activity of several copper-dependent enzymes can lead to a variety of symptoms such as connective tissue abnormalities, tortuosity of blood vessels and peculiar hair (kinky, steely hair or pili torti) [1,2]. The phenotypic features of MD can be divided into at least three categories: classical MD which leads to death in early childhood, the less severe atypical MD with longer survival, and the mildest allelic form Occipital Horn Syndrome (OHS). The neurological symptoms of OHS patients are milder than those found in patients with atypical MD and lead to a clinical picture mainly characterized by connective tissue manifestations. The majority of MD patients display the phenotype of classical MD, but milder phenotypes are seen in about 9% of the patients, and approximately one third of these have OHS [6].

To date, about 250 mutations in the *ATP7A *gene have been reported and are partly registered in the Human Mutation Database http://www.hgmd.org. Different types of mutations have been described: cytogenetic visible chromosome aberration, point mutations (deletions, insertions, missense and nonsense mutations, splice-site mutations), gross deletions including one or several exons [7], and more recently, exon duplications [8].

Traditional mutation-screening with PCR amplification of the coding region of *ATP7A *(23 exons) followed by sequencing, fails to detect exon duplications. In this study, we identified exon duplications in 20 patients using Multiplex ligation-dependent probe amplification (MLPA), and we have carried out detailed analyses of the *ATP7A *mRNA in 11 patients.

## Patients and Methods

### Patients

Thirty-three Menkes patients, in whom no mutation could be identified in the *ATP7A *gene (Genbank: NP_000043.2) either by sequencing or by exon PCR [9], were included in this study. The patients were referred to the Kennedy Center between 1994 and 2006 by a number of genetics centers in Europe and in USA for molecular confirmation of the diagnosis. All 33 patients had a well-documented clinical diagnosis of MD.

The patients (P7-P20) classified by the referring genetics centers as patients with classical MD, have symptoms such as hypothermia, feeding difficulties, convulsions, abnormal hair, dry skin, cutis laxa, hypopigmentation, bladder diverticula, cryptochidism, ataxia, mental retardation, decreased serum copper, decreased serum ceruloplasmin and abnormal radiographs. The patients typically died before they were 3 years old.

The patients (P2, P3, P4 and P5) classified by the referring genetics centers as patients with the atypical form of MD have fewer symptoms, are more attentive and interested in surroundings and survive longer. This group of patients typically survived for at least seven years.

One patient was classified by the referring genetics center as having the OHS phenotype (P1). He is at the time of writing 24 years old.

### Multiplex Ligation-dependent Probe Amplification

Genomic DNA was extracted from either leucocytes or cultured fibroblasts using standard methods. The MLPA ATP7A kit "SALSA MLPA P104" was obtained from MRC-Holland (Amsterdam, The Netherlands). The kit contains probes for 22 exons of the *ATP7A *gene (no probe was available against exon 23) as well as 11 control probes for other locations at the X-chromosome. Details on probe sequences can be found at the company's website http://www.mrc-holland.com. MLPA was performed according to the manufacturer's protocol. Reaction products were separated on an ABI model 310 capillary sequencer or an ABI3130XL sequencer (Applied Biosystems, Foster, CA) and GeneScan 3.1 software was used to size the PCR products and to obtain relative peak areas (RPA) as described previously [10]. The RPA of each probe was determined by dividing the peak area of each individual probe by the sum of the peak areas of all the control probes obtained for that sample. The RPA from each probe was then compared to that of a control sample by dividing the RPA with the RPA for the same probe obtained from a control sample, resulting in an RPA ratio. An RPA ratio of approximately 1 is normal whilst an RPA ratio of approximately 2 indicates a duplication of the exon.

### Polymorphism analysis

For patients whose polymorphic markers were known to be located in the duplicated fragments (from the size of the duplicated fragment or from verification by Q-PCR, see below), these markers were analyzed in order to establish whether the fragments originated from a single or from two different X-chromosomes. The regions containing the polymorphic STR, CA repeat, G00-437-244 located in intron 5 [11], the SNP Rs2227291 located in exon 10 [12], and a SNP in intron 13 (c. 2782-29C > A) [12] respectively, were amplified by PCR in relevant patients. The region containing the CA repeat was amplified using the primer-pair; F: 5'gccaagtattatgaagcaagg-3'/R: 5'-taccagtcttgaccccaaaca-3'. The region containing the SNP in exon 10 was amplified using the primer-pair; F: 5'-atatatgtgaatttcagcattttttaa-3'/R: 5'-atgtatttccaatgattggcc-3' and the region containing the SNP in intron 13 was amplified using the primer-pair; F: 5'--caccacacctggccattaac-3'/R: 5'-tcactctcccactccaaacc-3'. The PCR amplified fragments with the CA repeats were analyzed in an ABI 3130XL followed by detection with GeneMapper 3.0 Software (Applied Biosystems, Foster, CA). The SNPs were analyzed by sequencing the amplified PCR products.

### Q-PCR

Q-PCR was performed using genomic DNA (50 ng/sample). PCR amplification and detection was performed with an ABI7500 (Applied Biosystems) in accordance with the manufacturer's instruction using SYBR-Green. In patients P3, P6, and P7 the inclusion of the CA repeat in the duplicated region was confirmed by Q-PCR using primers located close to the position of the CA repeat. In contrast, Q-PCR revealed that the CA repeat was not included in the duplication in patient P11. As the primer-pair used for amplification of the CA repeat was not applicable for Q-PCR, the Q-PCR was performed using the primer-pairs; F: 5'-tggagggtgtaggaatgtatatgaaa-3'/R: 5'-tcaccttgcttcataatacttggcta-3' and F: 5'-atacaacccccaatgatagcaga-3'/R: 5'-ggctccaaatccaagttctcg-3' located upstream and downstream of the CA repeats, respectively.

### Cell cultures

Skin samples were collected from 11 patients, for diagnostic purposes. The fibroblasts were cultured as described previously [13].

### Characterization of *ATP7A *mRNA

Total RNA was isolated from approximately 5 × 10^6 ^cells of cultured skin fibroblasts with the RNAeasy Mini Kit (Qiagen). Single-stranded cDNA was synthesized with the High-Capacity cDNA Archive Kit (Applied Biosystems). The cDNA was used for PCR amplification with *ATP7A*-specific primer pairs flanking the duplication of interest. We amplified the duplicated region directly in 9 patients. The Advantage 2 PCR Kit (Clontech) was used for the amplification. The products were separated on a 1% agarose gel, and the fragments were excised and purified (Qiaquick gel extraction kit, Qiagen) before sequencing with the PCR amplification primers. For two patients, we performed RT-PCR on the duplication borders. For P10, who had a duplication of exon 3_17, we used the primers 16U and 4L and for P12, who had a duplication of exon 7_10, we used the primers 10U and 8L.

### Sequencing

The Big Dye Terminator v.3.1 Cycle Sequencing Kit was used for sequencing, and the products were analyzed in an ABI model 310 capillary sequencer.

### Quantitative RT-PCR

Real-time PCR was performed for relative quantification of the total amount of *ATP7A *transcript using probes located outside the duplicated regions. Real-time PCR amplification and detection were performed as described previously [14]. Real-time PCR was performed on cDNA obtained from patient- and control-fibroblasts. A Taq-Man 6-carboxy-fluorescein (FAM) labeled probe and primer pair against the boundary between exon 1 and exon 2 (part number Hs00921963_m1) in the *ATP7A *cDNA was used to detect *ATP7A *transcript. A FAM or VIC (Applied biosystems proprietary dye) labeled probe and primers for the human *GAPDH *transcript (part number 4352934E and 4326317E respectively) were used as an endogenous control. Relative quantification of *GAPDH *transcript was carried out on parallel samples. RNA from fibroblasts obtained from each of the patients was harvested two independent times. The cDNA samples obtained from the two RNA preparations were assayed in the concentrations; 100 ng/sample and 50 ng/sample respectively, in a total volume of 25 μl. Each dilution was assayed in triplicate. All probes were purchased from Applied Biosystems. PCR amplification and detection was performed with an ABI7300 or ABI7500 (Applied Biosystems) in accordance with the manufacturer's instruction. The threshold cycle (C_T_) is defined as the fractional cycle number at which the fluorescence passes a fixed threshold. Standard curves of (C_T_) values compared with log cDNA concentration were prepared by assaying five-fold serial dilutions of control cDNA, from 100 ng/sample to 0.16 ng/sample, with the *GAPDH *and *ATP7A *probes respectively.

## Results

**Figure 1:**
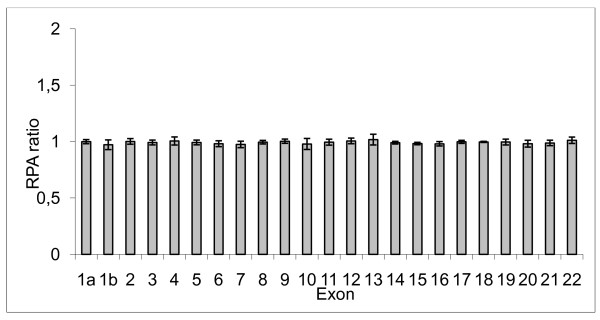
**Barchart of the MLPA results**. The results are presented as the mean RPA ratio of each exon, obtained from five healthy controls. The error bars represents the standard deviation.

Thirty-three Menkes patients were analyzed for duplications by MLPA. Duplication of two or more contiguous exons was found in 20 patients. The obtained results are shown in Table 1. The detected duplicated regions contained from 2 to 15 exons. Furthermore, results obtained from 5 control samples including standard deviation (SD) are illustrated in Figure 1. The phenotypes of the 20 patients are ranked as 1) severe classical form 2) milder atypical form with longer survival and 3) OHS. The phenotypes and MLPA results are summarized in Table 2. Notably, all patients with duplication of only two exons (and known phenotypes) displayed atypical MD or OHS, whereas the patients with duplication of a region with more than two exons all displayed classical MD. Unfortunately, the clinical phenotype is not known for patient P6 who had a duplication of two exons.

**Table 1:** RPA ratios for *ATP7A *probes in 20 patient DNA samples (P1-P20)

Exon/Patient	C	P1	P2	P3	P4	P5	P6	P7	P8	P9	P10	P11	P12	P13	P14	P15*	P16	P17	P18	P19	P20
Ex 1a	0.936	0.974	0.920	0.922	1.034	0.921	0.904	1.020	0.922	0.956	0.796	0.969	1.016	0.959	0.982	0.957	1.032	0.804	0.884	0.837	0.994

Ex 1b	0.934	0.970	0.903	0.945	0.938	0.953	1.010	1.061	0.903	0.991	0.856	1.030	1.015	0.943	0.998	0.943	0.904	1.032	0.851	0.960	0.959

Ex 2	1.005	0.992	1.014	1.071	1.014	1.045	0.971	1.006	0.968	0.991	0.859	1.001	1.012	1.002	1.030	0.908	0.974	0.885	0.983	1.000	0.963

Ex 3	0.954	**1.905**	0.975	1.042	1.021	1.006	1.012	**1.765**	0.861	**1.752**	**1.717**	0.973	1.021	0.946	1.017	0.985	0.999	0.969	0.924	0.979	0.954

Ex 4	1.028	**2.009**	1.024	1.155	1.052	1.009	1.076	**1.910**	1.088	**1.878**	**1.903**	0.848	1.033	0.956	0.997	0.956	0.995	1.125	1.024	0.979	1.002

Ex 5	0.990	0.944	**1.841**	1.011	0.971	1.014	1.082	**1.889**	**1.686**	**1.880**	**1.662**	0.915	0.932	0.952	1.170	0.946	0.904	1.030	0.948	0.987	0.948

Ex 6	0.953	0.964	**1.914**	**2.096**	0.934	0.939	**1.884**	1.000	**1.813**	**1.850**	**1.723**	**1.725**	0.983	0.949	1.030	0.961	1.012	0.941	0.882	1.009	0.933

Ex 7	1.017	0.931	0.988	**1.960**	0.943	1.011	**1.925**	0.981	**1.688**	**1.860**	**1.629**	**1.827**	**2.339**	**1.874**	**1.879**	**1.305**	1.011	0.946	0.912	0.971	0.935

Ex 8	0.950	1.050	1.000	0.748	1.014	1.015	1.034	0.990	1.010	**1.854**	**1.980**	**1.723**	**2.908**	**1.943**	**1.845**	**1.343**	**1.943**	**2.346**	**1.786**	**1.845**	**1.884**

Ex 9	0.990	1.049	1.035	1.057	1.021	1.026	1.032	0.970	1.062	**1.937**	**1.884**	**1.762**	**2.904**	**2.055**	**2.010**	**1.330**	**1.994**	**1.985**	**1.769**	**1.873**	**1.789**

Ex 10	0.903	1.020	1.031	0.872	1.003	0.968	0.993	1.023	0.977	**1.816**	**1.834**	**1.742**	**2.845**	**1.917**	**1.772**	**1.536**	**1.875**	**1.989**	**1.860**	**1.883**	**1.633**

Ex 11	1.031	0.979	1.024	1.000	1.004	0.970	1.094	1.024	1.058	**1.800**	**1.847**	**1.842**	0.990	**1.855**	**1.911**	**1.455**	**1.842**	**2.119**	**1.636**	**1.930**	**1.936**

Ex 12	1.060	1.032	1.032	1.069	1.070	1.013	1.037	1.000	1.014	**1.870**	**1.564**	**1.962**	1.032	**1.644**	**2.234**	**1.211**	**1.762**	**1.703**	**2.118**	**1.858**	**1.761**

Ex 13	1.058	1.068	1.041	1.148	**1.907**	0.855	1.104	1.095	0.988	**1.635**	**1.756**	**2.901**	1.025	1.014	1.199	**1.290**	1.034	0.909	**1.995**	**1.846**	**1.838**

Ex 14	1.000	0.989	1.010	0.978	**1.649**	1.034	1.184	1.005	0.979	0.907	**1.853**	**2.569**	0.949	0.973	1.196	**1.345**	0.942	1.203	**1.969**	**1.886**	**1.910**

Ex 15	1.040	1.026	0.988	0.806	1.024	0.938	1.031	0.972	1.008	0.950	**1.717**	**1.996**	1.022	0.981	1.042	0.995	1.088	0.940	1.053	**1.907**	**1.855**

Ex 16	0.998	1.095	1.043	1.031	1.050	**1.946**	1.066	0.967	0.980	0.990	**1.687**	0.943	1.054	1.075	0.995	1.075	1.102	0.905	0.862	1.029	**1.721**

Ex 17	0.997	0.990	0.965	0.963	1.003	**1.786**	1.007	0.993	0.958	0.975	**1.686**	0.834	0.964	0.960	1.042	0.920	1.012	0.926	0.930	0.990	**1.861**

Ex 18	0.994	1.017	0.972	1.019	0.978	0.964	1.160	0.984	0.991	0.936	0.949	0.874	1.002	1.009	0.922	1.009	0.984	1.120	0.915	0.999	0.942

Ex 19	0.964	1.035	1.030	1.128	1.036	0.983	0.061	1.003	1.041	1.006	0.934	0.904	1.045	1.008	1.088	1.008	1.073	0.946	0.962	0.990	0.904

Ex 20	0.976	0.957	0.991	0.825	0.981	0.986	0.912	0.990	0.979	0.917	0.921	0.918	1.008	1.018	1.057	0.925	0.993	0.980	0.729	0.993	0.924

Ex 21	0.977	1.054	1.035	0.866	1.066	1.011	1.001	0.959	0.961	0.950	0.905	0.910	1.044	1.064	1.120	1.064	1.003	0.928	0.775	0.995	0.957

Ex 22	0.968	0.963	0.927	1.059	0.985	0.930	1.154	0.994	0.974	1.002	0.907	0.952	0.970	0.949	1.164	0.904	0.959	1.072	1.152	0.956	0.945

RPA ratios for the *ATP7A *located MLPA probes in a control (C) and the 20 patient DNA samples (P1-P20). The MLPA kit include in addition 11 control probes (not shown in the figure) located at other positions of the X-chromosome.

*The mother of the index patient is tested.

**Table 2:** Exon duplications identified in the *ATP7A *gene

Patient (reference number)	Duplication:Mutation (identified on genomic DNA)	Phenotype	Mutation origin
P1 (95287)	Ex3_4dup: c.121-?_1336+?dup	OHS	Unknown

P2 (96220)	Ex5_6dup: c.1337-?_1707+?dup	Atypical	De novo

P3 (93250)	Ex6_7dup: c.1544-?_1869+?dup	Atypical	Mother carrier

P4 (9926)	Ex 13_14dup: c.2627-?_2916+?dup	Atypical	Mother carrier

P5 (96267)	Ex16_17dup: c.3112-?_3511+?dup	Atypical	Unknown

P6 (92251)	Ex6_7dup: c.1544-?_1869+?dup	Unknown clinical phenotype	Unknown

P7(95288)	Ex3_5dup: c.121-?_1543+?dup	Classical	Unknown

P8 (95265)	Ex5_7dup: c.1337-?_1869+?dup	Classical	Unknown

P9 (93226)	Ex3_13dup: c.121-?_2781+?dup	Classical	Mother carrier

P10 (96205)	Ex3_17dup: c.121-?_3511+?dup	Classical	De novo

P11 (94249)	Ex6_15dup: c.1544-?_3111+?dup	Classical	Mother carrier

P12 (94253)	Ex7_10 dup: c.1708-?_2406+?dup	Classical	Unknown

P13 (95245)	Ex7_12dup: c.1708-?_2626+?dup	Classical	Unknown

P14 (91211)	Ex7_12 dup: c.1708-?_2626+?dup	Classical	Unknown

P15 (93261)	Ex7_14 dup: c.1708-?_2916+?dup	Classical	Mother carrier

P16 (91212)	Ex8_12dup: c.1870-?_2626+?dup	Classical	Mother carrier

P17 (9620)	Ex 8_12 dup: c.1870-?_2626+?dup	Classical	Mother carrier

P18 (92238)	Ex8_14dup: c.1870-?_2916+?dup	Classical	Unknown

P19 (9322)	Ex8_15dup: c.1870-?_3111+?dup	Classical	De novo

P20 (96291)	Ex8_17dup: c.1870-?_3511+?dup	Classical	Mother carrier

Summary of exon duplications identified in the 20 patients.

### Source of the mutations

In order to test whether the observed duplications originated from two different X-chromosomes, polymorphism markers located in the duplicated regions were analyzed in selected patients and in the mothers of patients with de novo mutations; P2, P10, and P19 (Table 3 and Materials and Methods). None of the patients had different allelic variants at any of the investigated polymorphic sites. The mothers of P10 (3_17dup) and P19 (6_15dup) were both homozygous at the investigated SNPs. In contrast, the mother of P2 was found to be heterozygous at the CA repeat (Table 3).

**Table 3:** Investigation of polymorphism in the duplicated regions

Location	Polymorphism analysed	Type SNP/STR	
Intron 5	CA5 (G00-437-244) (Genome data base)	STR OH: (0.600)^#^	*Size of PCR fragment, copy 1/copy 2:* P2 (de novo): 176/176 P2 Mother: 176/180 (heterozygous) P3: 182/182 P6: 180/180 P7: 182/182 P8: 182/182

Exon 10	Rs2227291 (NCBI), c.2299 G > C	SNP OH: 0.599 (NCBI)	*Nuclotide at position c.2299:* P9: G/G P10 (de novo):G/G; P10 Mother: G/G (homozygous) P19 (de novo): G/G; P19 Mother: G/G (homozygous) P11: G/G P17: G/G P18: G/G P13, P14, P15, P16, and P20: All C/C

Intron 13	c.2782-29C > A (IVS13-29C > A)	SNP OH: Unknown	*Nucleotide at position c.2782-29:* P9: C/C P10 (de novo):C/C; P10 Mother: C/C (homozygous) P19 (de novo); P19 Mother: C/C (homozygous) P20: C/C P4, P11 and P15: All A/A

Selected patients (and their respective mothers) were investigated for polymorphism at a polymorphic CA repeat (STR) and at two polymorphic SNPs located in the duplicated regions. STR: short tandem repeat; SNP: single nucleotide polymorphism. Observed heterozygosity frequencies (OH). ^# ^ref [11].

### Characterization of the *ATP7A *transcripts in the patients

**Figure 2:**
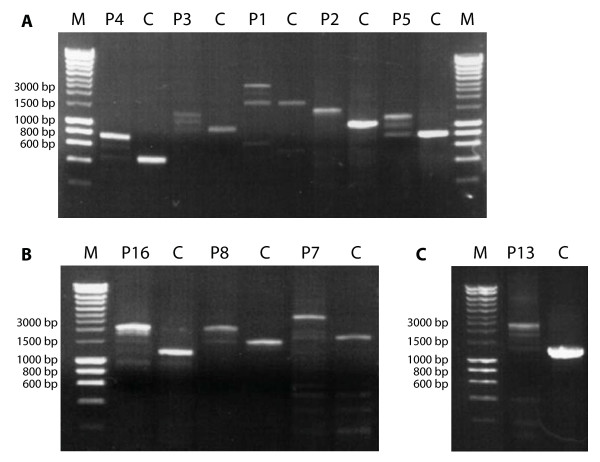
**Investigation of cDNA obtained from patients (P)**. A: Patients with duplication of 2 exons and atypical MD (P2; P3; P4 and P5) or OHS (P1) phenotypes. B and C: Patients with duplication of a region containing more than 2 exons and classical MD. The cDNA fragments expected to contain the duplicated exons were amplified by PCR (see Materials and Methods). The corresponding fragments from a control cell culture (C) were investigated in parallel. Hyperladder I was used as a marker (M).

The exact location and orientation of the duplicated exons were established by analyzing the *ATP7A *transcripts in cultured fibroblast from eleven selected patients. Fragments that spanned the individual duplications were amplified by RT-PCR using primer-pairs flanking the duplications (Table 4). RNA from five patients with a duplicated region containing only 2 exons (Figure 2A) and from four patients with duplication of more than two exons was investigated (Figure 2B, C). Several transcripts of different sizes were observed in all patients (Figure 2). The size of the largest transcript was in all patients, larger than the transcript obtained from control samples, and the difference in size corresponds to the expected size of the duplication. When sequencing the largest transcript in the 9 patients, we found -in all patients- that they contain the duplicated exons and that the duplicated part of the *ATP7A *gene was arranged in tandem, head to tail, with the original copy (Table 5). The duplicated exons were incorporated in the final *ATP7A *transcript at the normal splice sites, and in all 9 patients the duplication disrupted the translational reading frame. For two patients, RT-PCR analysis was carried out with special focus on the duplication borders. cDNA from P10 with a duplication of exon 3-17 was investigated with the primer-pair 16U/4L and cDNA from P12 with a duplication of exon 7-10 was investigated with the primer-pair 10U/8L (Table 4). Sequencing of the amplified fragments (P10: ex16-ex17-ex3-ex4, and P12: ex10-ex7-ex8) revealed that the duplications also in these two patients were in tandem head to tail (not shown). PCR amplification of control cDNA with the two primer pairs (16U/4L and 10U/8L), did as expected, not lead to the formation of any PCR product, as a borderline between exon 17 and exon 3 and a borderline between exon 10 and exon 7 only was present in P10 and P12, respectively.

**Table 4:** Primers used for spanning the duplicated cDNA fragments

Patient	Forward primer (5' to 3' direction)	Reverse primer (5' to 3' direction)
P1: Ex3_4dup	2U:atggatccaagtatgggtgtga	6L:tcacagtggctccaaatccaag

P2: Ex5_6dup	4U:caaaaagcagcccaagtacctc	7L:tattttatgtacgcaggaggc

P3: Ex6_7dup	5U:acacgaatgagccgttggtagt	10L:ggtggttgccagcacaatcagtacgtcc

P4: Ex13_14dup	12U:aggaggcaaatttccagtgga	15L:cagggacatgcaatacacagaactg

P5: Ex16_17dup	15U:tcccgaacagaaacgataatacga	19L:tctagctgttttactgttgtctccagt

P7: Ex3_5dup	2U:atggatccaagtatgggtgtga	7L:tattttatgtacgcaggaggc

P8: Ex5_7dup	4U:caaaaagcagcccaagtacctc	10L:ggtggttgccagcacaatcagtacgtcc

P13: Ex7_12dup	6U:gtgatagaaaatgctgatgaa	12L:ctggaaatttgcctcctggaact

P16: Ex8-12dup	7U:cctggcaaccaacaaagcaca	15L:ttcagcagttcccacaatgg

P10: Ex3_17dup	16U: ccattgtgggaactgctgaaagtaac	4L: cctttgctgtgacccttctg

P12: Ex7_10dup	10U: acgtactgattgtgctggcaac	8L: aagaccgtctccattgtcttattt

The sequence of primers used for spanning the duplicated cDNA fragments in selected patients.

**Table 5:** PCR products obtained by spanning

**Transcripts in patients with atypical MD or OHS**.		
**Patient**	**Transcripts**	**Predicted effect on reading frame**

P 1: Ex3_4dup	I: 2-3-4-3-4-5	I:Out of reading frame
	II:2-3-4-4-5	II: In reading frame
	III:2-3-4-5	III: wild-type

P2: Ex5_6dup	I: 4-5-6-5-6-7	I:Out of reading frame
	II:4-5-6-7	II: wild-type

P3: Ex6_7dup	I:5-6-7-6-7-8	I:Out of reading frame
	II:5-6-6-7-8	II:Out of reading frame
	III:5-6-7-8	III: wild-type

P 4: Ex13_14dup	I:12-13-14-13-14-15	I:Out of reading frame
	II:12-13-14-14-15	II:In reading frame
	III:12-13-14-15	III: wild-type

P5: Ex16_17dup	I:15-16-17-16-17-18	I:Out of reading frame
	II:15-16-16-17-18	II:In reading frame
	III:15-16-17-18	III: wild-type

Transcripts in patients with Classic MD.		

Patient	Transcripts	Predicted effect on reading frame

P7: Ex3_5dup	I: 2-3-4-5-3-4-5-6	I:Out of reading frame
	II:2-3-4-5-5-6	II: In reading frame
	III:2-3-4-5	III: wild-type

P8: Ex5_7dup	I: 4-5-6-7-5-6-7-8-9	I:Out of reading frame
	II:6-7-8-9	II: wild-type

P13: Ex7_12dup	I:7-8-9-10-11-12-7-8-9-10-11-12-13-14	I:Out of reading frame
	II: 7-8-9-10-8-9-10-11-12	II:In reading frame
	III: 7-8-9-10-11-12	III: wild-type

P16: Ex8_12dup	I:7-8-9-10-11-12-8-9-10-11-12-13-14	I:Out of reading frame
	II: 7-8-9-10-8-9-10-11-12-13-14	II:In reading frame
	III: 7-8-9-10-10-11-12-13-14	III:In reading frame
	IV: 7-8-9-10-11-12-13-14	IV: wild-type

The different PCR products were isolated from the gels shown in Figure 2, and sequenced. The various transcripts are indicated by roman numerals. Arabic numerals refer to the exons contained in the respective PCR products. The predicted effects on the reading frame are indicated.

In all patients a transcript of similar size as the wild-type *ATP7A *transcript was visible (Figure 2). In patient P1 with OHS, this product accounted for a substantial fraction of the total amount of transcript (Figure 2A). In the other patients a faint -in some cases almost invisible- band corresponding to the wild-type transcript could be observed (Figure 2). There was enough product to perform sequencing in all cases, and the sequencing confirmed that the bands indeed were wild-type transcript. Thus wild-type transcript is expressed in all patients. When sequencing PCR-amplified fractions of the different smaller transcripts obtained from the patients, we found that several exon-skipping events occurred within the duplicated regions (Table 5). In some splicing variants the reading frame was intact, while in others it was disrupted.

### Relative quantification of *ATP7A *transcript in patient cells

**Figure 3:**
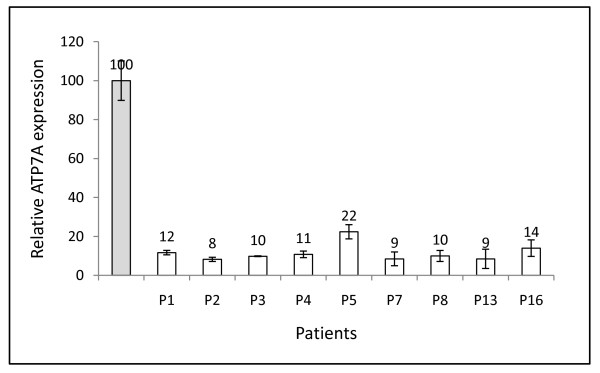
***ATP7A *mRNA expression in the patients**. Barchart showing the relative *ATP7A *mRNA expression in 9 patients, compared to control fibroblasts with a defined value of 100. The analysis has, for each patient, been performed on cDNAs from two different RNA preparations. The amount of *GAPDH *transcript was used as a normalization reference. The samples were analysed in triplicates. In all experiments, the amounts of *ATP7A *and *GAPDH *mRNA were calculated by linear regression of the lines generated by the standard curves; log cDNA concentration against C_T _(see Materials and Methods). The presented results show the mean value of the two cDNA preparations. Error bars represents the standard deviation of the results obtained from the two RNA preparations. The normalized *ATP7A*_N _value was calculated by dividing the *ATP7A *mRNA value by the *GAPDH *mRNA value in parallel samples. The value relative to unaffected control fibroblasts was calculated by dividing the normalized *ATP7A*_N _value from each patient by the normalized *ATP7A*_N _value obtained from control fibroblasts. The average ATP7A expression from fibroblasts from four independent healthy individuals (controls) is presented in the grey bar.

The ordinary RT-PCR (Figure 2) is not quantitative. Therefore, the relative amount of total *ATP7A *transcript was determined by real-time PCR with probes that recognized an exon-exon junction located outside the duplicated region. In order to normalize any differences in the cDNA-input in the samples, a probe that detected the housekeeping gene, *GAPDH*, was used as an endogenous control (Figure 3). The total amount of *ATP7A *transcript varied from 8% to 22% of the amount found in the normal control sample. The patient with the highest amount of transcript (22%) had atypical MD. Otherwise, there was no clear difference between the total amounts of transcripts in patients with OHS or atypical phenotypes, and patients with classical phenotypes. The total amount of *ATP7A *transcript is in all patients low relative to the healthy control, and is difficult to quantify exactly. The obtained C_T _values were between 29 and 31 indicating a copy number between 10^3 ^and 10^2 ^per sample.

## Discussion

MLPA was used to test for any evidence of duplications in the *ATP7A *gene in 33 Menkes patients in whom no mutation had been detected with standard diagnostic tools. Five controls were additionally assayed with the MLPA assay and the results showed very little variation between individuals (Figure 1). This in turn demonstrates the robustness of the MLPA technique in copy-number determination. We identified exon duplications in 20 of the patients. This corresponds to a duplication frequency of approximately 4% for the combined cohort of 468 independent Menkes patients with confirmed mutations in the *ATP7A *gene who has been referred to the Kennedy Center [15]. In comparison, the estimated frequency of deletions in the *ATP7A *gene in almost the same cohort of patients is 17% [6]. No mutation could be identified by MLPA in 13 of the investigated Menkes patients. It is possible that they contain a mutation in the regions of the *ATP7A *gene not investigated, such as the intronic sequences, the promoter or the 3'-UTR sequence. The copper excreting ability of fibroblast from 11 of the 13 males were analysed and the obtained results were in all cases in agreement with Menkes disease.

In three of the patients-P2, P10 and P19-the duplications seemed to be de novo mutations, only present in the somatic cells of the affected male (although germline mutations in the mother cannot be ruled out). Eight of the patients had inherited the duplication from the mother. It was not possible to obtain DNA from the mothers of the last 9 patients.

The presence of the same allelic variant of the G00-437-244 marker in P2 combined with the heterozygosity in the mother of P2, indicate that the duplicated fragment was derived from the same chromosome as a result of an intra-chromosomal event. How the duplications in the rest of the patients occurred is unknown. However, the presence of the same allelic polymorphic variant in the duplicated regions in all the investigated patients could suggest that the duplicated fragments in these patients as well, are derived from the same chromosome.

Several mechanisms that lead to duplications have been proposed for those identified in other diseases, such as Duchenne muscular dystrophy gene: homologous and non-homologous unequal chromatid exchange by recombination and synthesis-dependent non-homologous end joining [16,17]. In homologous recombination, sequence homology between the two parental DNA strands in the crossover is required for strand exchange. Homologous recombination could occur between repetitive elements in different introns of the gene.

The tandem duplications create one duplication junction flanked by two sequences which are normally separated in the genome. The junctions seem to be located at the border between two different intronic sequences; the intronic sequence downstream of the first copy and the intronic sequence upstream of the second copy of the duplicated fragment. We found that 40% (8/20) of the *ATP7A *duplication breakpoints are located in intron 7. Furthermore, 25% (5/20) of the duplication breakpoints are located in intron 6 and another 25% (5/20) are located in intron 12. In total, 12 different introns are involved in the duplications presented here, and in all cases, the two involved introns have repeat regions in common. Using the online bio-informatics tool http://zeus2.itb.cnr.it/cgi-bin/wwwrepeat.pl we found that the majority of these repeats are of the Alu or SVA type. Interestingly, introns 3, 8, and 20 do not contain any repeats, and there are no duplication junctions in any of these introns. As the duplication junctions have not been sequenced in order to verify whether there is any significant sequence homology around the junctions or if any nucleotides are inserted at the junction, it is unknown how the duplications in *ATP7A *occur. Further characterization of the duplication junctions might reveal the mechanisms.

RT-PCR results from 9 selected MD patients revealed that all the *ATP7A *transcripts that contain the duplications are out of frame, which leads to the formation of premature termination codons. None of these transcripts are expected to encode functional ATP7A protein, but are probably degraded by the nonsense-mediated decay (NMD) mechanism [18]. This concurs with the reduced amount of transcript observed in all patients (Figure 3).

Patient P1, with an Ex3_4dup is diagnosed with OHS. Until now about 14 patients with OHS have been reported [6,8,19]. We have previously identified an Ex3_4del in a patient with MD with unexpectedly mild symptoms and long survival. The mutated transcript in this patient contains a premature termination codon after only 46 codons. We verified that the mild phenotype was due to the synthesis of, at least partially, functional Menkes protein as a result of re-initiation at internal ATG codons located in exon 5 [14]. As the transcript in P1 does not contain a premature termination codon until codon 458, it is unlikely that reinitiation takes place in this patient. It is more likely that the OHS-phenotype in P1 is attributed to a relatively large amount of wild-type transcript. P1 has approximately 12% total *ATP7A *transcript relative to the control level, and according to Figure 2, a relatively large fraction of this is wild-type transcript.

Wild-type transcript is observed in all patients. However, the exact amount of wild-type transcript is difficult to determine. We have previously shown that 2-5% wild-type transcript relative to the control level is sufficient to allow the development of the mildest phenotype-OHS [13]. It is possible that even less transcript is sufficient to permit the development of atypical MD. Thus, it is possible that at least a fraction of the other patients with duplication of only two exons have atypical MD phenotypes because of the presence of a small amount of wild-type transcript. Recently, four duplications in the *ATP7A *gene, identified in MD patients, have been published: Ex3_5dup, Ex2_4dup, Ex8_12dup and Ex8-17dup [8]. The four duplications were all identified in patients with classical MD [8]. Three of the four duplications, Ex3_5dup, Ex8_12dup and Ex8-17dup were also identified in our cohort of patients.

## Conclusions

In conclusion, we demonstrate that duplications in the *ATP7A *gene were organized in tandem, in a head to tail direction, and we suggest that the development of OHS and the atypical MD phenotype is at least partly due to the fact that the splicing apparatus by-passes the duplicated region, leading to the production of wild-type transcript.

## List of abbreviations

MD: Menkes disease; NMD: nonsense-mediated decay; TGN: trans-Golgi network; OHS: Occipital Horn Syndrome; MLPA: Multiplex ligation-dependent probe amplification; RPA: relative peak areas.

## Competing interests

The authors declare that they have no competing interests.

## Authors' contributions

Generation and analysis of the clinical data: HK, KS, NH, LBM. Mutation identification and Real-Time PCR: MM, LBM, TS. Investigation of polymorphisms and Q-PCR: LBM. RT-PCR: MM. Study concept: LBM. Manuscript draft: LBM. Substantial participation in the design of the project and writing the paper: LBM, TS, NH. All authors participated in the writing of this paper in the context of their individual expertise, and all have read and approved the final version of the article.
